# Causal role of medial superior frontal cortex on enhancing neural information flow and self-agency judgments in the self-agency network

**DOI:** 10.1016/j.neuroimage.2025.121245

**Published:** 2025-04-28

**Authors:** Yingxin Jia, Kiwamu Kudo, Namasvi Jariwala, Phiroz Tarapore, Srikantan Nagarajan, Karuna Subramaniam

**Affiliations:** a Department of Psychiatry, University of California, San Francisco, CA, USA; b Medical Imaging Center, Ricoh Company Ltd., Kanazawa, Japan; c Department of Clinical Psychology, Palo Alto University, Palo Alto, CA, USA; d Department of Neurological Surgery, University of California, San Francisco, San Francisco, CA, USA; e Department of Radiology and Biomedical Imaging, University of California, San Francisco, San Francisco, CA, USA

**Keywords:** Self-agency, Reality monitoring, Magnetoencephalography imaging, Medial frontal cortex, Repetitive transcranial magnetic stimulation, Neural information flow

## Abstract

Self-agency is being aware of oneself as the agent of one’s thoughts and actions. Self-agency is necessary for successful interactions with the outside world (reality-monitoring). Prior research has shown that the medial superior prefrontal gyri (mPFC/SFG) may represent one neural correlate underlying self-agency judgments. However, the causal relationship remains unknown. Here, we applied high-frequency 10 Hz repetitive transcranial magnetic stimulation (rTMS) to modulate the excitability of the mPFC/SFG site that we have previously shown to mediate self-agency. For the first time, we delineate *causal* neural mechanisms, revealing precisely how rTMS modulates SFG excitability and impacts *directional* neural information flow in the self-agency network by implementing innovative magnetoencephalography (MEG) phase-transfer entropy (PTE) metrics, measured from pre-to-post rTMS. We found that, compared to control rTMS, enhancing SFG excitability by rTMS induced significant increases in information flow between SFG and specific cingulate and paracentral regions in the self-agency network in delta-theta, alpha, and gamma bands, which predicted improved self-agency judgments. This is the first multimodal imaging study in which we implement MEG PTE metrics of 5D imaging of space, frequency and time, to provide cutting-edge analyses of the *causal* neural mechanisms of how rTMS enhances SFG excitability and improves neural information flow between distinct regions in the self-agency network to potentiate improved self-agency judgments. Our findings provide a novel perspective for investigating *causal* neural mechanisms underlying self-agency and create a path towards developing novel neuromodulation interventions to improve self-agency that will be particularly useful for patients with psychosis who exhibit severe impairments in self-agency.

## 1. Introduction

Self-agency is the awareness of oneself as the agent of one’s thoughts and actions. Self-agency is of cardinal importance because it is integral to self-awareness in the context of human interactions with the outside world (i.e., reality monitoring) (Subramaniam, 2021; Haggard, 2017). Reality monitoring is the ability to differentiate internal self-generated information from externally-derived information. In our prior reality monitoring studies, in which subjects distinguish self-generated from externally-derived information, participants showed increased medial superior prefrontal gyri (mPFC/SFG) activity during the successful encoding and retrieval of self-generated information, which correlated with accurate judgments of self-agency (i.e., accurate identification of self-generated information), indicating this mPFC/SFG site represents one neural correlate of self-agency (Subramaniam, 2021; Subramaniam et al., 2019; Subramaniam et al., 2012).

The mPFC/SFG site represents a critical brain region that consistently exhibits heightened activity before self-generated actions (that do not occur prior to externally-perceived actions). This increased mPFC/SFG self-preparatory activity which is thought to lead to the experience of self-agency, has been observed across convergent imaging studies (functional MRI, magnetoencephalography (MEG), and electroencephalography (EEG)) as well as single neuron studies (Subramaniam et al., 2019; Subramaniam et al., 2020; Passingham et al., 2010; Cabeza and Jacques, 2007; Keller and Heckhausen, 1990; Khalighinejad et al., 2018; Fried et al., 2011). Given these correlative data that mPFC/SFG supports self-agency in healthy adults, we now test whether enhancing medial SFG excitability can *causally* modulate self-agency during a reality-monitoring task.

In the present double-blinded mechanistic study, for the first time we use repetitive transcranial magnetic stimulation (rTMS) as a causal neurostimulation tool to test whether increasing medial SFG excitability with high-frequency 10 Hz rTMS, will improve directed neural information flow in the self-agency network to enhance self-agency judgments during reality monitoring. Participants were assigned to either active rTMS to enhance medial SFG excitability or 10 Hz rTMS applied to a control distal site outside the self-agency network (*N* = 15). To-date, the neural mechanisms as to how rTMS modulates the excitability of a targeted region, such as SFG, and induces neural plasticity via trans-synaptic propagation of neural information flow to its connected sites within the self-agency network, remain a mystery.

In this study, we capitalize on the powerful linkage of multimodal MRI/TMS/MEG that enables us to localize both spatial aspects of TMS with regard to each subject’s neuroanatomical MRI, and move beyond correlative paradigms to delineate with MEG the *causal* neurophysiological temporal and frequency band characteristics of medial SFG stimulation with respect to control TMS. Specifically, we use the high spatiotemporal resolution of MEG by implementing cutting-edge MEG phase-transfer entropy (PTE) analyses, measured from pre-to-post rTMS, to quantify how much information in the future of a region-of-interest (ROI) target is predictable when knowing the past state of the neural source (Engels et al., 2017; Hillebrand et al., 2016; Rivolta et al., 2015; Jia et al., 2022). MEG PTE metrics thus enable *causal* computations of the temporal propagation in *directed* neural information flow, measured not only in different MEG frequency spectra but with millisecond timing resolution, between the stimulated SFG region and connected regions in the self-agency network that is induced by active rTMS to medial SFG, compared to baseline and control rTMS. Taken together, here, we employ a causal multimodal imaging framework to delineate, for the first time, the precise neural mechanisms as to how enhancing medial SFG excitability with rTMS modulates the temporal propagation of directed neural information flow between distinct regions in the self-agency network to potentiate improved self-agency judgments.

During the default mode, when participants are at rest and left to think to themselves, the lower frequency band oscillations such as delta-theta and alpha bands represent an resting-state rhythm associated with self-generated internal thought processes that underlie self-agency, and are dominant in medial frontal and adjacent cingulate cortex during rest across MEG, EEG and fMRI studies (Jann et al., 2009; Mantini et al., 2007; Nunez, 1981; Samogin et al., 2019). We also know rTMS propagates trans-synaptically, and that SFG has direct connections with the cingulate gyrus (CG) and premotor/supplementary motor cortex (i.e., the paracentral lobule, PCL) in the self-agency network (Catani et al., 2012; Zhang et al., 2012). In addition, convergent functional imaging studies across fMRI, PET and EEG studies, have shown increased neural activity in CG and PCL, immediately prior to subjects engaging in self-generated actions, compared to externally-triggered actions, which is thought to reflect subjects’ internal thoughts and the volition in motor preparation to translate these thoughts to initiate self-generated actions that results in the experience of self-agency (Haggard, 2017; Khalighinejad et al., 2018; Doganci et al., 2023; Buckner et al., 2008). This neural activity increase in medial frontal activation extending to CG and PCL activation, shown in beta and gamma activity, is thought to mediate self-predictions in ‘self-generated forward models’ (also known as efference copies of the motor command sent to sensory cortices) that model the expected sensory outcome of self-generated actions, that lead to the experience of self-agency (Subramaniam, 2021; Subramaniam et al., 2019; Chang et al., 2016; Franken et al., 2018; Niziolek et al., 2013; Ranasinghe et al., 2019). Overall, these findings suggest that medial frontal activation and the temporal propagation of neural connectivity and information flow to cingulate and paracentral lobule are critical nodes in the self-agency network that function to improve self-prediction mechanisms that lead to a unitary experience of self-agency.

Taken these prior findings together, we hypothesized that compared to control rTMS, enhancing medial frontal excitability by rTMS would improve trans-synaptic neural plasticity in the self-agency network manifested as increased neural information flow from medial SFG to PCL and CG in either delta-theta (2–8 Hz), alpha (8–12 Hz), beta (12–30 Hz), or gamma (30–50 Hz) frequency bands, and this improved neural information flow would predict improved self-agency judgments. If these hypotheses are confirmed, this would be the first study in which we implement state-of-the-art MEG PTE techniques, measured from pre-to post rTMS, to delineate the precise causal neural mechanisms underlying self-agency by revealing how enhancing medial SFG excitability by rTMS improves neural information flow between distinct regions in the self-agency network to potentiate improved self-agency judgments.

## 2. Results

**Fig. 1.**
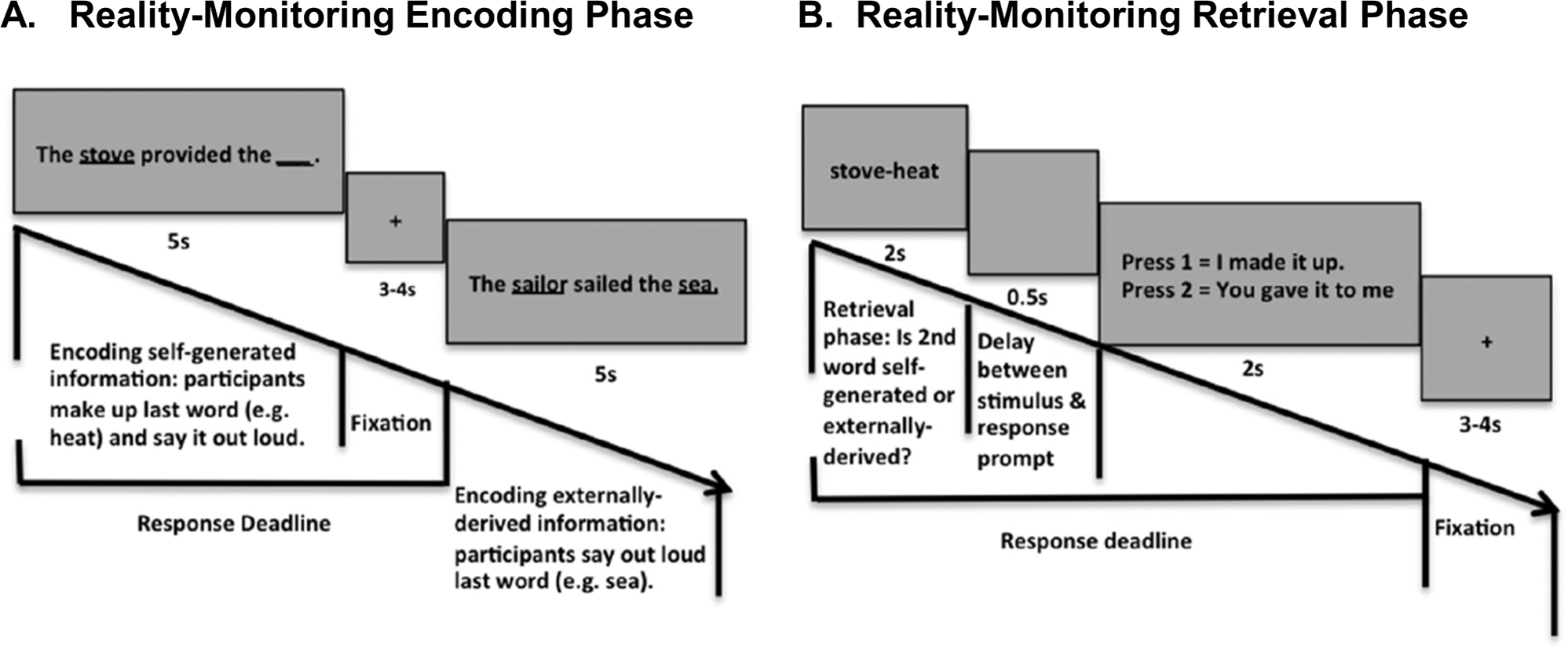
Reality Monitoring Task Design. A. During the encoding phase, for half the sentences, the final word was either left blank for participants to make up themselves (e.g., The stove provided the __) or was externally-given by the experimenter (e.g., The sailor sailed the sea). B. During the retrieval phase, participants were randomly presented with the noun pairs from the sentences (e.g., stove-heat), and had to identify with a button-press whether the second word was previously self-generated or externally-derived.

**Fig. 2.**
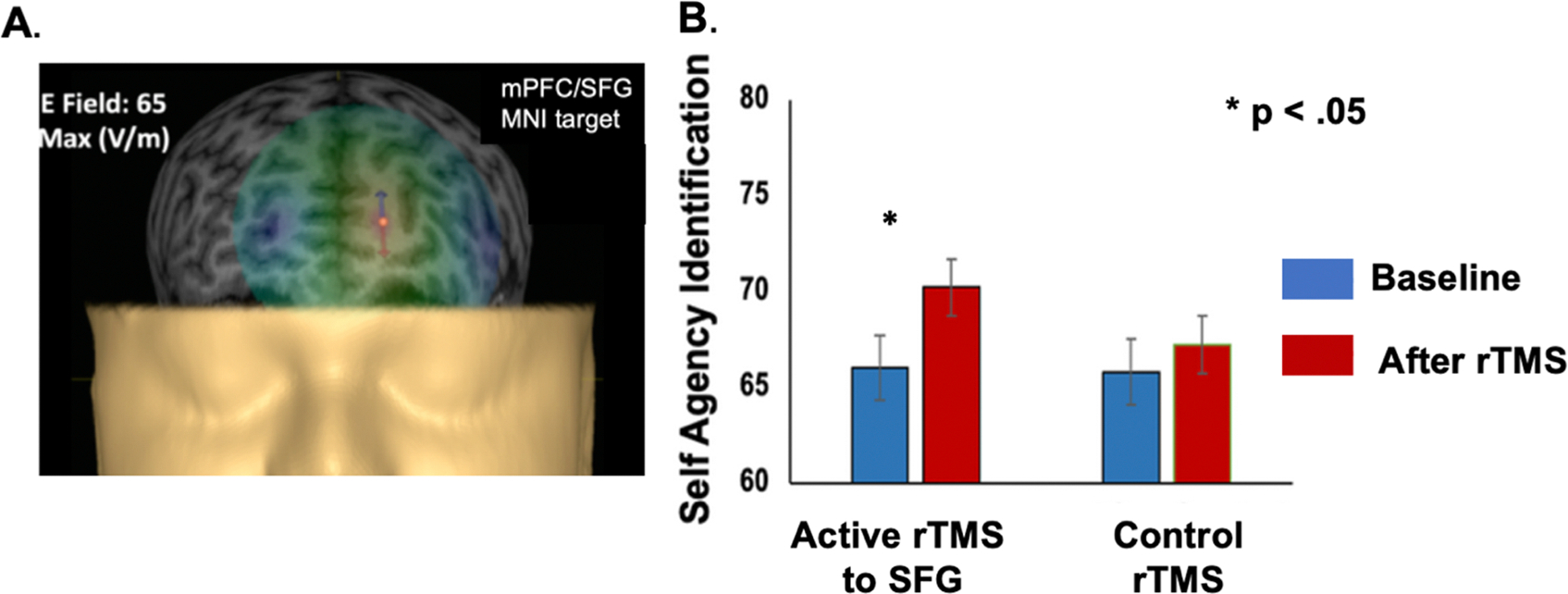
A. Illustration of example subject’s head model, depicting the E-field strength in real-time during active high-frequency 10 Hz rTMS to medial SFG target site (MNI:x,y,*z*=−8,56,15), defined by the functional overlap of medial frontal activity underlying self-agency across our prior convergent neuroimaging fMRI and MEG studies (Subramaniam et al., 2019; Subramaniam et al., 2012). B. Paired *t*-tests revealed participants had significant improvement in self-agency judgments (i.e., % accuracy for identifying self-generated information) that was observed only after participants completed active rTMS to SFG, but not found in the control rTMS condition, compared to baseline. Error bars represent standard error of the mean.

**Fig. 3.**
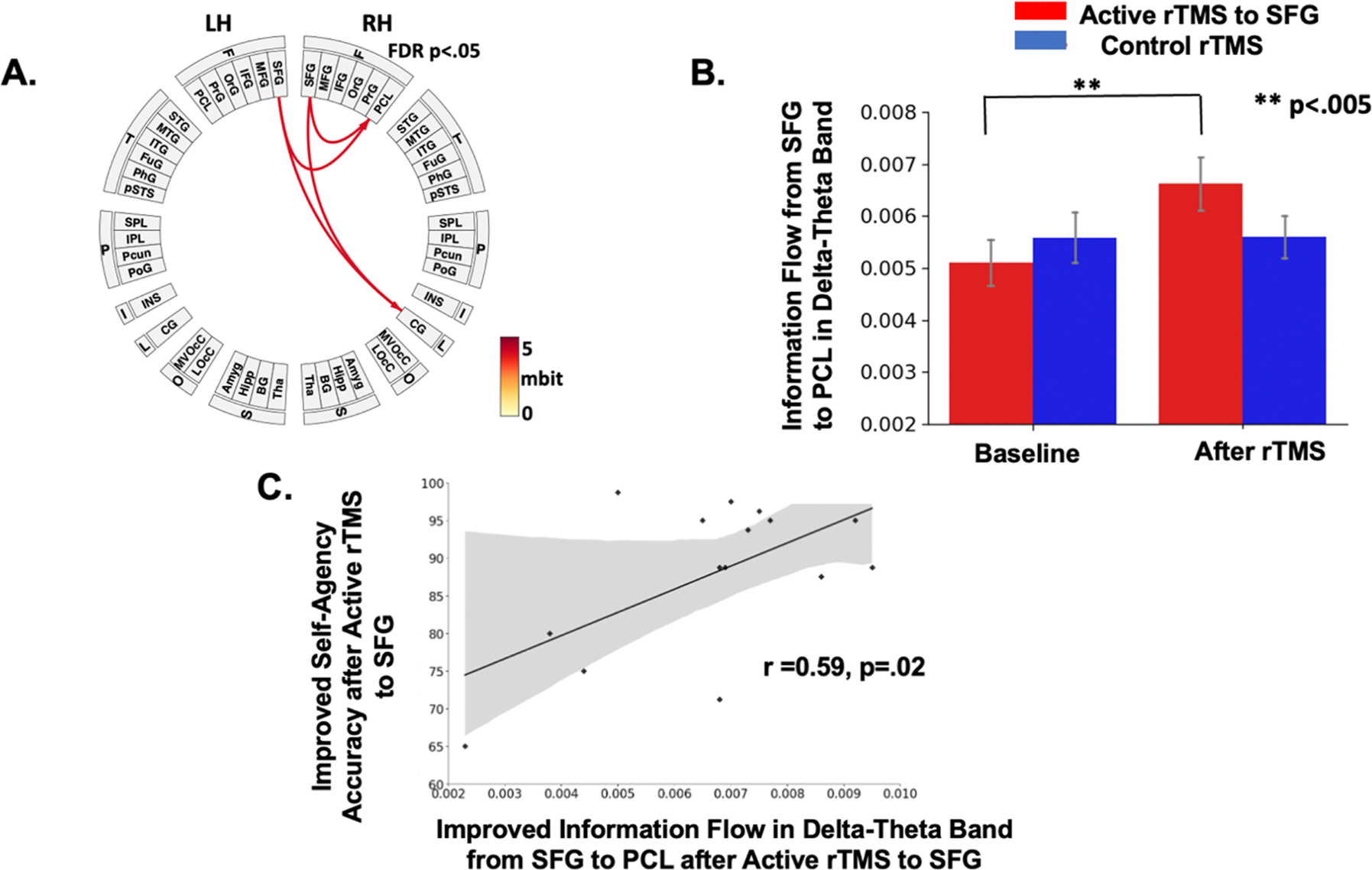
Paired *t*-tests revealed that enhancing SFG excitability by rTMS induced increased neural information flow (mbit units) from SFG to cingulate gyrus (CG) and paracentral lobule (PCL) in the self-agency network in delta-theta frequency band (2–8 Hz), compared to baseline, shown by: (A) Brainnetome atlas-based connectogram, and (B) bar plots. Error bars represent standard error of the mean. (C) Increased information flow from SFG to PCL in delta-theta band predicted improved self-agency judgments on the reality-monitoring task (i.e., % accuracy for identifying self-generated information) after rTMS compared to baseline, only in the group who completed rTMS applied to SFG. *LH=left hemisphere, RH=right hemisphere.

**Fig. 4.**
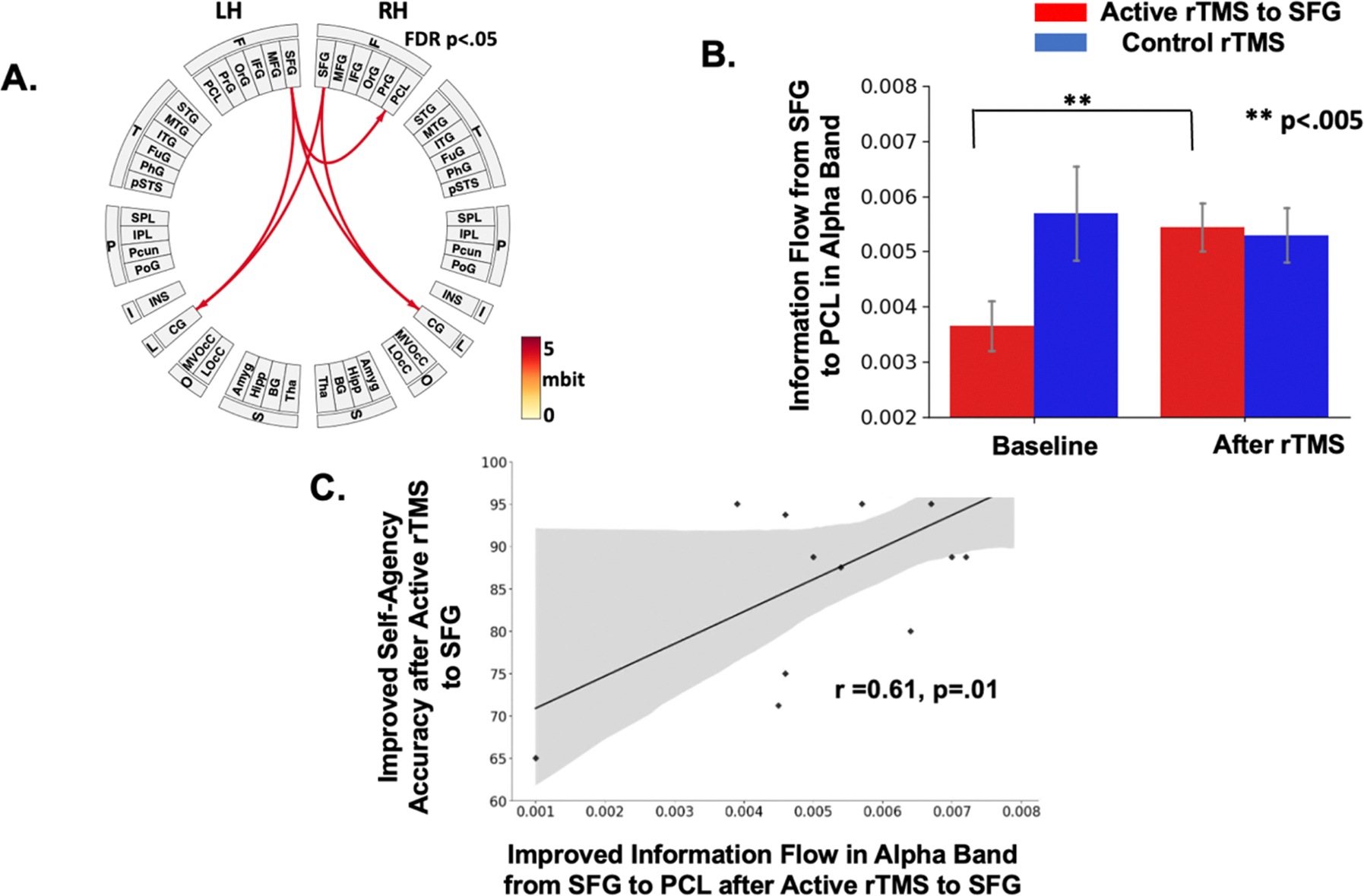
Paired *t*-tests revealed that enhancing SFG excitability by rTMS induced increased neural information flow (mbit units) from SFG to CG and PCL in the self-agency network in alpha frequency band (8–12 Hz), compared to baseline, shown by: (A) Brainnetome atlas-based connectogram, and (B) bar plots. Error bars represent standard error of the mean. (C) Increased information flow from SFG to PCL in alpha band predicted improved self-agency judgments on the reality-monitoring task, after rTMS compared to baseline, only in the group who completed rTMS applied to SFG.

**Fig. 5.**
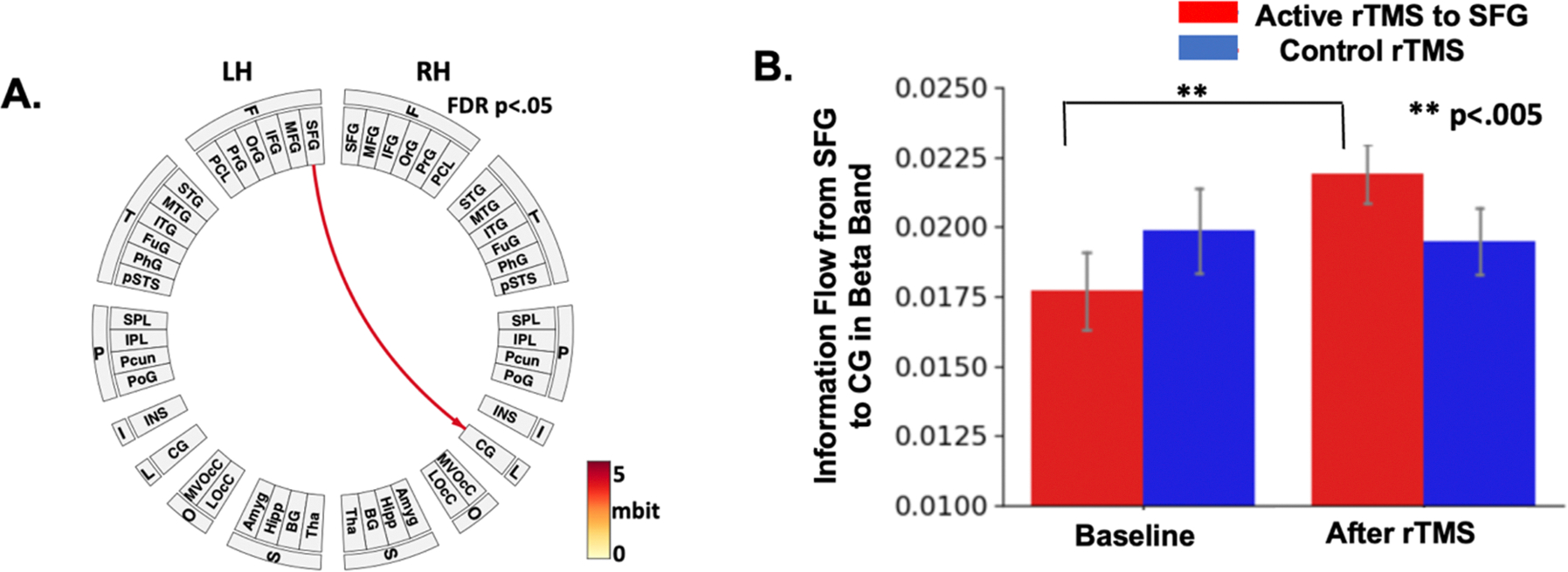
Paired *t*-tests revealed that enhancing SFG excitability by rTMS induced increased information flow (mbit units) from SFG to CG in the self-agency network in beta frequency band (12–30 Hz), compared to baseline, shown by: (A) Brainnetome atlas-based connectogram and (B) bar plots. Error bars represent standard error of the mean.

**Fig. 6.**
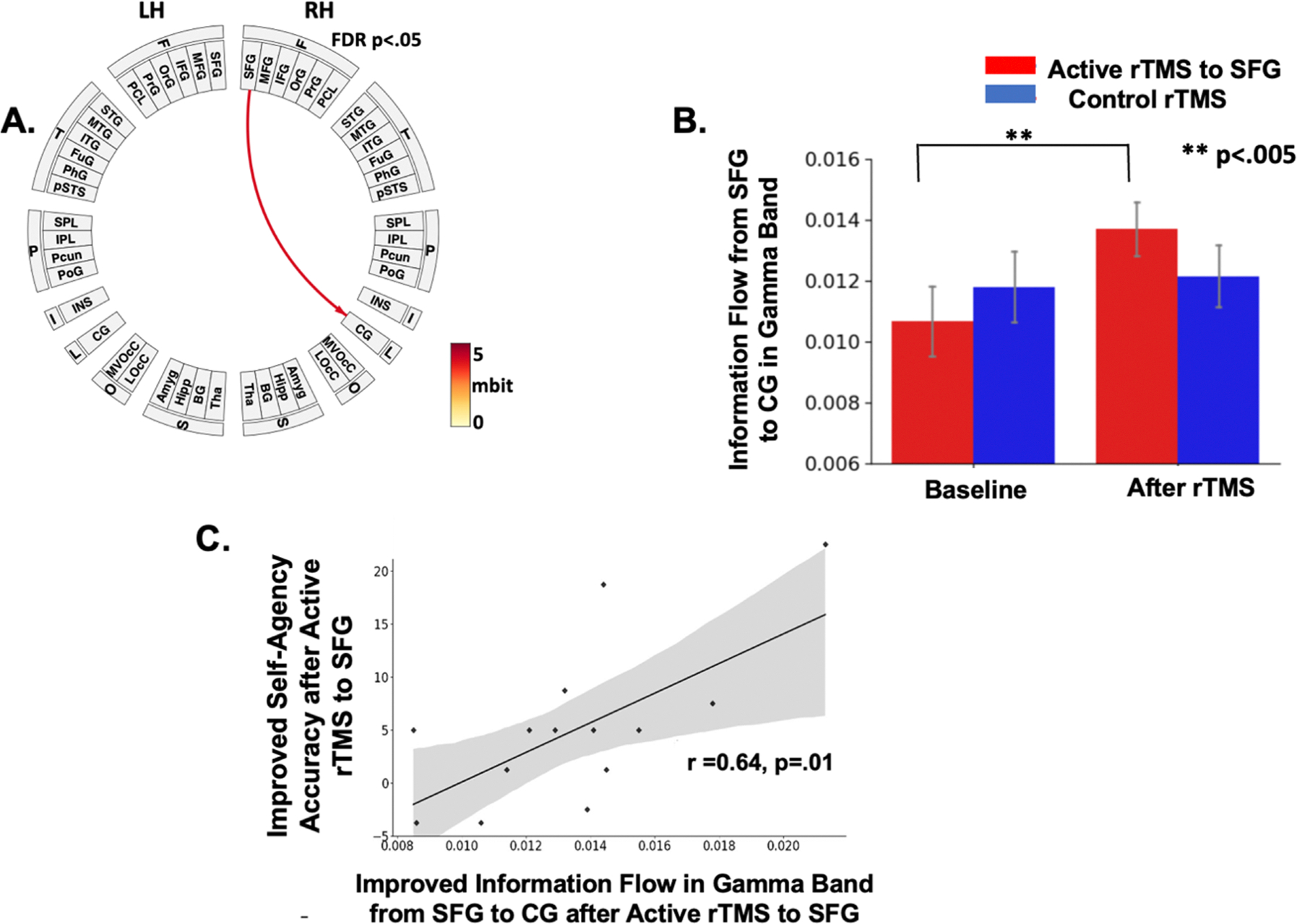
Paired *t*-tests revealed that enhancing SFG excitability by rTMS induced increased neural information flow (mbit units) from SFG to CG in the self-agency network in gamma frequency band (30–50 Hz), compared to baseline, shown by: (A) Brainnetome atlas-based connectogram and (B) bar plots. Error bars represent standard error of the mean. (C) Increased information flow from SFG to CG in gamma band predicted improved self-agency judgments on the reality-monitoring task after rTMS compared to baseline, only in participants who completed rTMS applied to SFG.

Consistent with our hypothesis, on the reality monitoring task (Fig. 1), repeated-measures ANOVA revealed that enhancing SFG excitability by rTMS induced significant improvement in self-agency judgments, compared to baseline (*F* = 7.7, *p* = 0.02). We did not find any difference in self-agency judgments in participants who completed the control rTMS condition, compared to baseline (*F* = 2.8, *p* = 0.12) (Figs. 2A–B). To delineate the neural mechanisms underlying these self-agency improvements, whole-brain repeated-measures 2 × 2 ANOVA revealed significant group (active rTMS of SFG vs. control rTMS of temporoparietal site) × time (baseline vs. post-rTMS) interactions, which revealed increased information flow from SFG to distinct regions in the self-agency network (such as the cingulate gyrus and paracentral lobule) in delta-theta (2–8 Hz) [*F* = 6.76, *p* = 0.01], alpha (8–12 Hz) [*F* = 6.28, *p* = 0.02], beta (12–30 Hz) [*F* = 7.99, *p* = 0.01], and gamma (30–50 Hz) frequency bands [*F* = 5.98, *p* = 0.02], which were significant at strict FDR thresholds, corrected for whole-brain multiple comparisons (FDR, *p* < 0.05). Subsequent simple-effects planned comparison paired *t*-tests revealed that the group by time interactions revealing increased information flow from SFG to cingulate gyrus (CG) and paracentral lobule (PCL) was specifically driven by active rTMS of SFG, compared to baseline, in delta-theta (2–8 Hz) [*t* = 3.33, *p* < 0.005], alpha (8–12 Hz) *t* = 3.47, *p* = 0.004], beta (12–30 Hz) [*t* = 3.29, *p* < 0.005], and gamma (30–50 Hz) frequency bands [*t* = 3.64, *p* < 0.003] (see Figs. 3–6).

Specifically, compared to control rTMS, regression analyses revealed that increased information flow from SFG to PCL was observed in lower frequency bands such as delta-theta and alpha bands, whereas increased information flow from SFG to CG was observed in higher gamma frequencies, which predicted improved self-agency judgments on the reality-monitoring task only in participants who completed rTMS targeting the SFG (see Figs. 3C, 4C, and 6C). In other words, enhancing SFG excitability by rTMS increased information outflow from SFG to CG in all frequency bands; however, increase in information outflow to CG predicted better self-agency judgments in higher-frequency gamma bands while increase in information outflow from SFG to PCL predicted better self-agency judgments in lower-frequency delta-theta and alpha bands.

Paired *t*-tests did not reveal improved self-agency judgments on the reality-monitoring task or increased information flow in any regions in participants who completed rTMS to the control left temporoparietal site, compared to baseline (all p’s >0.05). Overall, our findings provide the first causal evidence to show that the improvements in self-agency judgments were specifically due to the effects of medial SFG stimulation, and are unbiased by placebo or general stimulation effects from the control rTMS condition. Taken together, the present MEG findings, quantified by PTE metrics, provide the high spatiotemporal resolution to accurately capture with millisecond precision the neural changes in information flow that are induced by rTMS to SFG, and delineate for the first time the causal neurophysiological mechanisms of SFG stimulation on modulating the self-agency network.

## 3. Discussion

This study provides the first comprehensive demonstration, revealing that rTMS applied to medial SFG not only induced targeted modulation of SFG excitability but also induced trans-synaptic neural plasticity by stimulating increased neural information flow from SFG to distinct sites in the self-agency network, such as PCL and CG. Enhanced neural information flow from SFG to PCL and CG predicted and potentiated improved self-agency judgements on the reality-monitory task. Given that rTMS was applied to medial SFG in the active rTMS condition, we found rTMS effects shown by increased neural information flow in both left and right SFG as we had expected. In particular, increased information flow from SFG to PCL was observed in lower frequency bands (i.e., delta-theta and alpha), whereas increased information flow from SFG to CG was found in higher gamma frequencies, which predicted improved self-agency judgments on the reality-monitoring task. Importantly, the findings of increased neural information flow from SFG to PCL and CG that potentiated improved self-agency judgments, were only observed in the participants who completed rTMS targeting SFG but not in the control rTMS group. After participants completed control rTMS, we did not find any regions that showed increased neural information flow or improved self-agency judgments on the reality-monitoring task. Together, these convergent findings provide robust evidence that the increases in neural information flow from SFG to specific CG and PCL regions in the self-agency network that potentiated improved self-agency judgments were specific to rTMS modulation of SFG excitability, and are unbiased by placebo or general stimulation effects from the control rTMS condition.

The medial superior frontal cortex extending to cingulate gyrus is a central hub within the default mode network (DMN), which supports self-reflection and internal thought processes associated with self-agency (Jann et al., 2009; Mantini et al., 2007; Nunez, 1981; Buckner et al., 2008). Prior MEG, EEG, and fMRI studies have consistently shown that lower frequency band oscillations in delta-theta and alpha bands in medial frontal regions represent an idling rhythm during resting states that underlie self-agency (Jann et al., 2009; Mantini et al., 2007; Nunez, 1981; Samogin et al., 2019; Buckner et al., 2008). We have previously found across MEG and fMRI studies increased medial frontal neural activity extending to cingulate activation, not only while participants self-reflect during rest, but also during explicit reality-monitoring tasks when subjects were required to make self-agency judgments (Subramaniam, 2021; Subramaniam et al., 2019; Subramaniam et al., 2012; Subramaniam et al., 2016). Specifically, we found increased medial frontal neural activity extending to cingulate activation during the successful encoding and memory retrieval of self-generated information, which correlated with accurate judgments of self-agency, indicating that this medial frontal site represents a neural correlate of self-agency (Subramaniam, 2021; Subramaniam et al., 2019). We have also previously shown that this site can be directly modulated with rTMS to induce significant improvement in self-agency judgments during reality-monitoring (Subramaniam et al., 2020). In the present study, we now replicate and extend our prior findings (Subramaniam et al., 2020) in a different group of participants by implementing whole-brain MEG PTE metrics, measured from pre-to-post rTMS. MEG PTE computations enable us to move beyond traditional functional connectivity coherence-based analyses (which examine neuronal *correlations* or synchrony between regions) to a causal model in which we delineate here the precise mechanisms of how enhancing SFG excitability by high-frequency rTMS modulates *directed* neural information flow with millisecond precision from SFG to distinct regions in PCL and CG in specific frequency-bands to enhance neuronal communication that lead to the experience of self-agency.

Our results are consistent with prior studies that have shown increased neural activity in mPFC, extending to regions such as CG and PCL, in lower frequency alpha and theta bands, which is thought to reflect feedforward predictive coding models that model the expected sensory outcome of self-generated actions to enable the initiation of self-regulated goal-directed actions (Cao et al., 2017; Enriquez-Geppert et al., 2014). This increased neural activity in mPFC, extending to CG and PCL, has been shown to occur consistently prior to self-generated (but not prior to externally-perceived actions), indicating these regions are important for mediating self-predictions that model the expected sensory outcome of self-generated actions, and reflect the volition in motor preparation to translate these self-predictions to initiate self-generated actions that results in the experience of self-agency (Haggard, 2017; Khalighinejad et al., 2018; Doganci et al., 2023; Buckner et al., 2008; Chang et al., 2016; Franken et al., 2018; Cao et al., 2017). We have previously found increased medial frontal activation extending to cingulate activation, shown in beta and gamma activity, during self-prediction mechanisms on different tasks of reality-monitoring and speech monitoring, that results in the unitary experience of self-agency (Subramaniam, 2021; Subramaniam et al., 2019; Ranasinghe et al., 2019).

In summary, this is the first multimodal study in which we implemented whole-brain MEG neural source reconstructions that were used for our PTE analyses for each frequency band in each participant, assayed from pre-to-post rTMS. The fact that we found consistent results across all separate four bands provides conclusive evidence to validate the findings that SFG plays a critical role in self-agency, in that rTMS applied to SFG induced increased neural information flow from SFG to CG and PCL in each band that resulted in improved self-agency judgments. These neural and self-agency improvements were only observed in the active rTMS group and are unbiased by placebo or general stimulation effects from the control rTMS condition. Such causal approaches of applying innovative MEG PTE metrics are also quick and easy to implement in clinical settings as they only require participants to complete 4 min resting-state scans, measured from pre-to-post rTMS, thus facilitating the development of rapid and effective ways of measuring neural plasticity and treatment response to new neuromodulation treatment targets. By contrast, precision medicine TMS-fMRI research can only focus on the spatial domain (rather than temporal and frequency spectra domains). Moreover, as a result of the limited temporal resolution over several seconds, fMRI approaches are unable to capture neural processes with high temporal resolution which are critical for measuring fast fluctuations in neuronal oscillations that underlie agency (Subramaniam et al., 2019; Khalighinejad et al., 2018; Franken et al., 2018) and aberrations in neural oscillations that underlie agency impairments that result in psychosis (Liddle et al., 2016; Moran et al., 2019; Donati et al., 2021; Reulbach et al., 2007; Lee et al., 2006). This represents a significant barrier to developing precision-medicine TMS-fMRI treatments as networks underlying agency operate in the millisecond scale. In conclusion, the present multimodal imaging study using TMS/MEG/MRI provide the first cutting-edge evidence for delineating *causal* neural mechanisms underlying self-agency with millisecond-level precision, and create a path towards developing new neuromodulation interventions to improve self-agency that will be particularly useful for patients with psychosis disorders who exhibit severe impairments in self-agency.

## 4. Materials and methods

### 4.1. Participants and procedures

In the present double-blind randomized controlled trial, 30 healthy adults (20 males, 10 females, mean age = 42.7 years, mean education = 16.8 years) volunteered to participate in the MEG study at the University of California San Francisco (UCSF) (Table 1). All methods and procedures were performed in accordance with the guidelines of the Internal Review Board (IRB) at UCSF and were approved by the IRB at UCSF. Participants were recruited through our clinicaltrial.gov site (NCT04807530) or from our previous clinical studies in schizophrenia if they had consented to be contacted for future studies. Participants were evaluated by a clinical psychologist and completed questionnaires to meet the inclusion criteria. Inclusion criteria were no Axis I or Axis II psychiatric disorder (SCID—Nonpatient edition), no current or history of substance dependence or abuse, meets MRI criteria, good general physical health, age between 18 and 64 years, right-handed, and English as the first language. Participants gave written informed consent for this protocol and then completed an MEG resting-state scan and cognitive and clinical assessments at baseline. Participants were matched at a group level on age, gender, and education, and then randomly assigned to either the active 10 Hz rTMS condition targeting SFG or the control 10 Hz rTMS condition targeting the temporoparietal site outside the self-agency network. Immediately after the rTMS session, participants completed the MEG resting-state scan and the reality-monitoring task.

### 4.2. MRI protocol

Participants underwent T1-weighted imaging in a 3T Siemens scanner. Whole-brain structural MRI data were acquired using the following parameters (3Tesla, 3D sequence, flip angle 9°, TE 2.98 ms, TR 2300 ms, TI 900 ms, FOV 256 × 256 mm, matrix 256 × 256 × 252, NEX = 0.5, voxel dimensions 1 × 1 × 1 mm, slice thickness=1, slices per tab=160, acquisition time = 4:54 min).

### 4.3. MEG data acquisition

Each participant underwent four minutes of continuous resting-state recording inside a magnetically shielded room with a 275-channel whole-head MEG system (MEG International Services Ltd., Coquitlam, British Columbia, Canada) consisting of 275 axial gradiometers. Participants were instructed to keep their eyes closed and stay awake in a supine position during the MEG scans (sampling rate = 1.2KHz). All subjects verified that they were awake throughout the resting-state scans.

To provide anatomical head models for MEG analysis, a high-resolution 3D T1-weighted whole-brain magnetic resonance imaging (MRI) was acquired for each subject using a 3T Siemens scanner. For each subject, the outline of the brain on the structural scans was extracted, and the segmented brain was treated as a volume conductor model for the source reconstruction described below. Three fiducial coils (nasion, left and right preauricular points) were placed to localize the position of the head relative to the MEG sensor array. MEG coregistration with the structural MRI for each participant was performed based on three fiducial coil positions (nasion and left and right preauricular).

### 4.4. MEG data processing

The first step in data processing was to down-sample all the raw data to 1000 Hz, and remove cardiac, muscle and eye-twitch artifacts using independent component analysis. For each subject, a consecutive 4-min signal was digitally filtered using a bandpass filter of 0.7–150 Hz. Noisy channels which contained artifact due to head motion were removed based on visual inspection of the data. In addition, dual signal subspace projection (DSSP) (Sekihara et al., 2016) was applied to the filtered sensor signal to remove environmental noise using lead field vectors computed with an individual head model. Finally, Zapline noise filtering was applied to remove power line noise between 60 and 120 Hz (de Cheveigne, 2020).

After preprocessing the MEG data, whole-brain source reconstruction was implemented. For source reconstruction, isotropic voxels (5 mm) were generated from a template MRI, resulting in 15,457 voxels. The generated voxels were then warped to an individual head model. For each voxel, individual magnetic lead field vectors were computed in a forward model with single-shell model approximation (Nolte, 2003). The voxels for each participant were then indexed to 48 modules defined in the Brainnetome atlas (Fan et al., 2016). We used these 48 Brainnetome atlas modules as our ROIs.

To obtain whole-brain source-localized activity, an array-gain scalar beamforming approach (Sekihara et al., 2004) was applied to the 4-minute resting-state sensor time series. Beamformer weights were computed in the time domain, and the data covariance matrix for beamforming was calculated for the 4-minute sensor-time series. The applied beamforming provided voxel-level source timecourses on the 5-mm volumetric grids in the brain. For each brain module from the Brainnetome atlas, the representative regional timecourse was extracted by applying principal component analysis to the voxel timecourses within each module and taking its first principal component. Before the connectivity metrics were calculated, the 48 representative timecourses were filtered into different frequency bands: delta-theta (2–8 Hz), alpha (8–12 Hz), beta (12–30 Hz), and gamma (30–50 Hz) bands. Computations of source reconstruction and connectivity metrics were performed using MATLAB.

### 4.5. Phase-transfer entropy

Phase-transfer entropy (PTE) was used to evaluate pairwise directional interactions between region-of-interest (ROI) timecourses. PTE metrics compute the direction of information flow from source signal X to a target signal Y based on the phase time series (Lobier et al., 2014; Palus and Stefanovska, 2003). The phase timecourses were extracted from the ROI timecourses using Hilbert transform and were used to estimate pairwise directional interactions in neural information flow during resting-state scans. The PTE from source phase timecourse X to target phase timecourse Y as derived in Engels (2017) (Engels et al., 2017) is shown below:

(1) $$PTE_{X\to Y}=l\left(T_{t+\delta t};X_{t}|Y_{t}\right)=\sum_{t}p\left(Y_{t+\delta t},Y_{t},X_{t}\right){log}_{2}\left(\frac{p\left(T_{t+\delta t};Y_{t}|X_{t}\right)}{p\left(Y_{t+\delta t}|Y\right)}\right)$$

where δt is a delay in time, p(X,Y) is a joint probability, p(X|Y) is conditional probability and I(Y;X|Z) denotes the conditional mutual information between Y and X conditioned with Z. For PTE computations between all ROIs, the source signal X represents the phase timeseries of one ROI sending phase information, and the target signal Y denotes the phase time series of another ROI receiving the phase information.

To remove bias, an estimate of PTE was also made for shuffled data which creates a null distribution, shown below:

(2) $${\mathrm{PTE}}_{X\to Y}\mathrm{shuffled}=l(T_{t+\delta t};X_{t}^{\mathrm{shuffled}}|Y_{t})$$

The shuffle estimate was repeated 20 times by randomizing the order of the time points for each source ROI phase time, and the shuffled PTE estimate was computed as an average of the results obtained from these 20 trials. The shuffled PTE data estimate was then subtracted from the original PTE computation to provide further statistical corrections for significance by minimizing false positives (Lee et al., 2016):

(3) $$PTE_{X\to Y}=l\left(T_{t+\delta t};X_{t}|Y_{t}\right)-l(T_{t+\delta t};X_{t}^{\mathrm{shuffled}}|Y_{t})$$

PTE for all pairwise ROIs was expressed in the form of a matrix. Since there are 48 Brainnetome regions across the brain, the dimension of the PTE matrix was 48 × 48. The regional PTEs (vectors of regional measures) can be computed by averaging values across the components of the PTE matrix along the target (y) array and source (x) array dimensions. The regional PTEs were defined as follows:

(4) $$PTE_{\mathrm{out}}=\frac{1}{n_{ROI}-1}\sum_{y}\;PTE_{X\to Y}$$

and

(5) $$PTE_{in}=\frac{1}{n_{ROI}-1}\sum_{X}\;PTE_{X\to Y}$$

where $n_{ROI}$ is the number of ROIs (i.e., 48 in this case). $PTE_{\mathrm{out}}$ results from averaging across the target array and indicates the regional information outflow at a source brain region; while $PTE_{\mathrm{in}}$ results from averaging across the source array and denotes the regional information inflow at a target brain region.

For group-level analyses, we performed repeated-measures ANOVAs to compare changes in information flow, quantified by the PTE metrics (mbit units) after rTMS vs. baseline, in the active rTMS group compared to the control rTMS group. To minimize Type I error, statistical significance was based on strict whole-brain multiple comparison FDR corrections (FDR *p* < 0.05). Subsequent simple-effects planned comparison paired *t*-tests were used to compare changes in information flow in each group after rTMS, compared to baseline. Regression linear analyses were used to predict improved self-agency judgments (i.e., % accuracy for identifying self-generated information) based on changes in directional neural information flow (mbit units) in each group after rTMS compared to baseline. We also minimized Type II errors associated with correcting for multiple regression tests, by examining regressions only in pairs that showed significant changes in neural information flow that were induced by rTMS in each group, compared to baseline (FDR *p* < 0.05).

These MEG PTE calculations enable causal computations of changes in directed neural information flow between brain regions that is induced by rTMS, measured at the millisecond level. Using MEG PTE metrics, assayed from baseline to post-rTMS, here, we establish the precise neural mechanisms of how rTMS targeting SFG impacts the temporal propagation of neural communication in the self-agency network, compared to the control rTMS condition.

## 5. Reality monitoring task

All participants completed a reality monitoring task at baseline and immediately after the rTMS session (Fig. 1). As described in previous studies (Subramaniam et al., 2019; Subramaniam et al., 2012; Subramaniam et al., 2020; Subramaniam et al., 2018), the reality monitoring-task entails an encoding phase and a memory retrieval phase. All the participants completed eight runs, with 20 trials per run, totaling 160 trials for the whole task. During the encoding phase, participants were visually presented with semantically-constrained sentences with “noun-verb-noun” structures. On half the sentences, the final word was either left blank for subjects to make up on their own (i.e., *The* *stove* *provided the*), or was externally-given by the experimenter (i.e., *The* *sailor* *sailed the* *sea*). For each sentence, participants were told to pay attention to the underlined nouns for a subsequent memory test and to vocalize only the final word of each sentence. After the encoding phase, participants then completed the memory retrieval phase where they were randomly presented with the underlined noun pairs from the sentences (e.g., *sailor-sea*), and were asked to identify whether the second word was previously self-generated or externally-derived using a button box. At each time point, the sentences were completely different, containing different sets of matched semantically-constrained sentences. For each subject, accuracy for self-agency identification was computed as the percentage of correctly-identified self-generated items out of the total number of self-generated trials on the reality-monitoring task. Repeated-measures ANOVAs were implemented to examine group differences in self-agency judgments after rTMS compared to baseline.

## 6. rTMS protocol

Our TMS system is a state-of-the-art Nexstim Neuronavigated Brain Stimulator (Helsinki, Finland). This system integrates the TMS figure-8 coil with a navigational system with several landmarks on each subject’s scalp surface matched to their anatomical locations on the 3D MRI that allows for highly accurate cortical targeting individualized to each subject’s MRI anatomy. Once the targeted stimulation site is selected, the system calculates the strength of the electric field in real-time that is incident on the targeted cortical site (Petrov et al., 2017; Ruohonen and Ilmoniemi, 1999). The specific site that we target in subjects assigned to the active rTMS is based on our prior functional localization of medial superior frontal activity mediating self-agency (i.e., accurate identification of self-generated information) during reality monitoring across our convergent fMRI and MEG studies, labeled as the SFG ROI, on the Brainnetome atlas (Subramaniam et al., 2019; Subramaniam et al., 2012) (Fig. 2A). We have previously shown that this region can be directly modulated with rTMS to induce significant improvement in self-agency abilities (Subramaniam et al., 2020). For example, the electric field strength is shown as 65V/m in real-time that is applied to the targeted medial frontal site during rTMS in an example subject (Fig. 2A). In our previous study (Subramaniam et al., 2020), we had established the optimal rTMS dosage parameters that maximized tolerability/comfort in the present study. The present rTMS parameters have also been deemed safe by the rTMS Consensus Guidelines. The rTMS session consisted of 120 trains of 20 pulses (2 s duration of 10 Hz) for 20 mins at 110 % Resting Motor Threshold based on hand electromyography (Subramaniam et al., 2020), which we and others have previously shown to maximize safety and efficacy, without a single adverse event (Bentwich et al., 2011; Lee et al., 2016; Rabey et al., 2013). Due to prior reports of more discomfort and pain with higher-frequencies from our prior pilot study (Subramaniam et al., 2020), we did not use frequencies of >10 Hz or theta-burst stimulation, or longer protocols (single train durations >2 s).

## Supplementary Material

SupplementaryMaterials

Supplementary material associated with this article can be found, in the online version, at doi:10.1016/j.neuroimage.2025.121245.

## Figures and Tables

**Table 1:** Demographics (mean, SD) of Study Participants.

	Active rTMS to SFG	Control rTMS	p value

**Age (years)**	41 (16)	44 (18)	.60
**Gender**	10 M, 5F	10 M, 5F	1.0
**Education (years)**	17 (2.2)	17 (1.8)	.86

## Data Availability

Data will be made available on request.
