# Supplementary material for: Causal role of medial superior frontal cortex on enhancing neural information flow and self-agency judgments in the self-agency network
Source: Neuroimage. Author manuscript; Available in PMC 2026 Jun 23. (PMC13290074; doi:10.1016/j.neuroimage.2025.121245)
Supplement: SupplementaryMaterials [file NIHMS2182043-supplement-SupplementaryMaterials.docx]

**
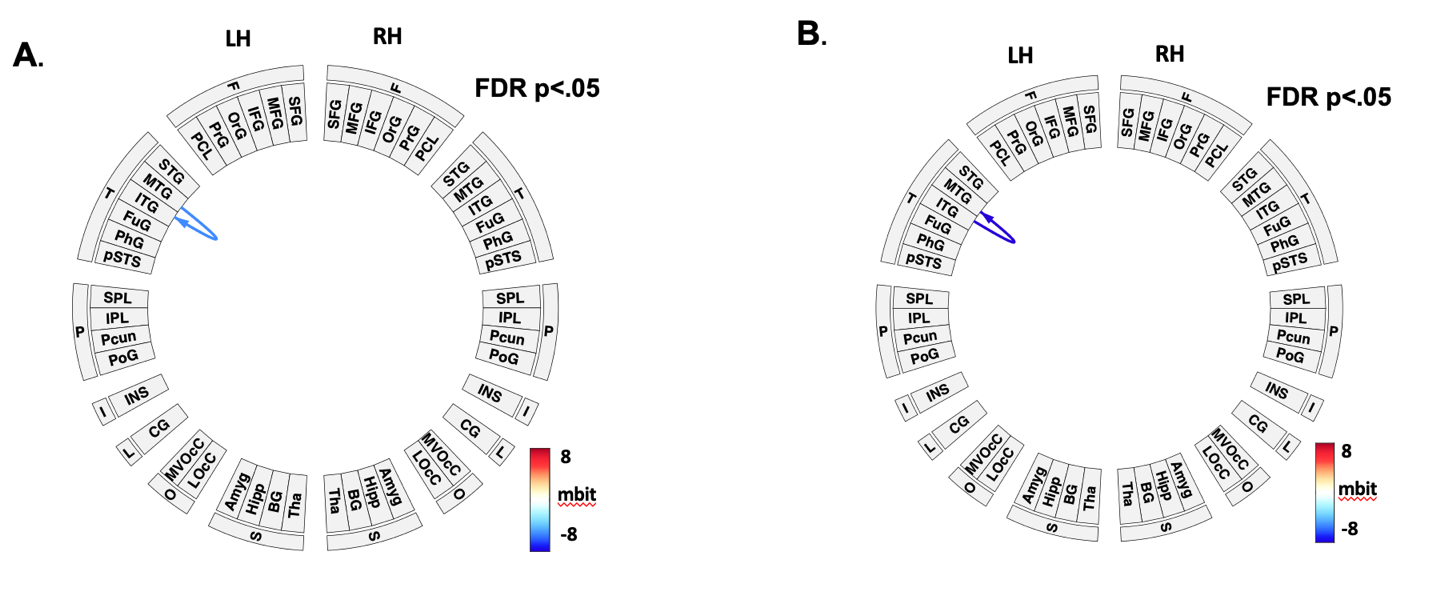
Supplementary Fig. 1.** No regions revealed increased information flow in participants who completed rTMS to the control temporoparietal site, compared to baseline. Paired t-tests revealed reduced information flow between middle temporal gyrus (MTG) and inferior temporal gyrus (ITG) in **(A)** alpha band and **(B)** beta frequency bands in participants who completed control rTMS, compared to baseline.
